# Long Non-coding RNA *IRAIN* Inhibits VEGFA Expression via Enhancing Its DNA Methylation Leading to Tumor Suppression in Renal Carcinoma

**DOI:** 10.3389/fonc.2020.01082

**Published:** 2020-09-02

**Authors:** Yang Li, Qingyang Luo, Zun Li, Yun Wang, Chaoyang Zhu, Tieqiang Li, Xiaodong Li

**Affiliations:** Department of Urinary Surgery, Huaihe Hospital, Henan University, Kaifeng, China

**Keywords:** renal carcinoma, long non-coding RNA *IRAIN*, vascular endothelial growth factor A, DNA methyltransferase, DNA methylation, tumor-suppressive potential, tumor development, tumor progression

## Abstract

**Aims:** Long non-coding RNA *IRAIN* (lncRNA *IRAIN*) plays a critical role in numerous malignancies. However, the function of lncRNA *IRAIN* in renal carcinoma (RC) remains enigmatic. The purpose of this study is to characterize the effects of lncRNA *IRAIN* on RC progression.

**Methods:** The expression pattern of lncRNA *IRAIN* and the vascular endothelial growth factor A (VEGFA) in RC tissues and cells was characterized by RT-qPCR and Western blot analysis. The roles of lncRNA *IRAIN* and VEGFA in the progression of RC were studied by gain- or loss-of-function experiments. Bioinformatics data analysis was used to predict CpG islands in the *VEGFA* promoter region. MSP was applied to detect the level of DNA methylation in RC cells. The interaction between lncRNA *IRAIN* and VEGFA was identified by RNA immunoprecipitation and RNA-protein pull down assays. Recruitment of DNA methyltransferases (Dnmt) to the *VEGFA* promoter region was achieved by chromatin immunoprecipitation. The subcellular localization of lncRNA *IRAIN* was detected by fractionation of nuclear and cytoplasmic RNA. Cell viability was investigated by CCK-8 assay, cell migration was tested by transwell migration assay, and apoptosis was analyzed by flow cytometry. The expression of epithelial–mesenchymal transition-related and apoptotic factors was evaluated by Western blot analysis. Finally, the effect of the lncRNA *IRAIN*/VEGFA axis was confirmed in an *in vivo* tumor xenograft model.

**Results:** LncRNA *IRAIN* was poorly expressed in RC tissues and cells with a primary localization in the nucleus, while VEGFA was highly expressed. Overexpression of lncRNA *IRAIN* or knockdown of *VEGFA* inhibited cell proliferation and migration and induced the apoptosis of RC cells. Bioinformatics analysis indicated the presence of CpG islands in the *VEGFA* promoter region. Lack of methylation at specific sites in the *VEGFA* promoter region was detected through MSP assay. We found that lncRNA *IRAIN* was able to inhibit VEGFA expression through recruitment of Dnmt1, Dnmt3a, and Dnmt3b to the *VEGFA* promoter region. LncRNA *IRAIN* was also able to suppress RC tumor growth *via* repression of VEGFA in an *in vivo* mouse xenograft model.

**Conclusion:** Our data shows that by downregulating *VEGFA* expression in RC, the lncRNA *IRAIN* has tumor-suppressive potential.

## Introduction

Renal carcinoma (RC) comprised several histological subtypes, including clear cells, type 1 papillary carcinoma, and type 2 papillary carcinoma (1), each subtype being characterized by a unique molecular morphology (2). Renal cell carcinoma (RCC) is present in more than 90% of all kidney cancers (3), with the most common histological subtype, clear cell RCC (ccRCC), appearing in 70–80% of RCC cases (4). Risk factors such as smoking, obesity, and high blood pressure contribute to the occurrence of RC (5). However, the underlying molecular mechanisms leading to the progression of RC are largely unknown.

Long non-coding RNA (lncRNA) transcripts typically exceed 200 nucleotides and are found in both the cytoplasm and the nucleus (6) where they regulate various biological processes (7). Various lncRNAs are known to have pro-tumorigenic or tumor-suppressing effects in RC (8). Interestingly, a previous study identified both oncogenic and anti-oncogenic roles for *IRAIN*, an lncRNA involved in a variety of cancers including breast cancer (9), non-small cell lung cancer (10), and pancreatic cancer (11). According to our bioinformatics predictions, lncRNA *IRAIN* targets the vascular endothelial growth factor A (VEGFA), which provides a better understanding of how IRAIN exerts its function.

VEGF is well-known as a major driver of angiogenesis and vascular permeability (12). As a latent tumor angiogenic gene, *VEGFA* is responsible for the induction of new blood vessels which bring oxygen and nutrients to the tumor microenvironment (13), playing a key role in tumor proliferation and metastasis (14). Of note, anti-angiogenic therapy in cancer using VEGF inhibitors has been an effective strategy for the treatment of RC (15) and metastatic RCC (16). Therefore, our study aims to investigate the specific effect of VEGF as a potential therapeutic target in RC.

Epigenetic reprogramming like DNA methylation and post-translational histone modifications in cancer cells leads to changes in the expression of genes which regulate tumor phenotypes (17). DNA methylation is oftentimes associated with cancer development (18) and consists of histone modifications, particularly histone H3 lysine 4 methylation (H3K4me) and H3K9 methylation (19). Previous studies found that alterations of VEGFC by s-adenosylmethionine-medicated methylation impeded progression of gastric cancer (20). Accordingly, we propose that lncRNA *IRAIN* could regulate VEGFA expression through methylation of its promoter region, thereby affecting the progression of RC. Our study will shed light on the functional role of lncRNA *IRAIN*, which may provide new strategies for the clinical treatment of RC.

## Materials and Methods

### Ethical Statement

The study was conducted with the approval of the Ethics Committee of Huaihe Hospital, Henan University. Informed consent forms were available from all participants, or their guardians, in studies involving human subjects. Experiments involving animal subjects were performed in accordance with the recommendations detailed in the Guide for the Care and Use of Laboratory Animals of the National Institutes of Health.

### Study Subjects

In this study, 40 RC patients hospitalized in Huaihe Hospital, Henan University, from January 2012 to January 2014 were recruited, including 22 males and 18 females (aged 42–76 years with a mean age of 55.35 ± 9.37 years). All patients were confirmed as RC positive by post-operative pathology in Huaihe Hospital, Henan University, had complete clinical records, complied with tumor-node-metastasis (TNM) staging standards, and voluntarily participated in the study and signed the informed consent form. None of the patients had recurrence and distant metastasis after treatment or history of mental illness or was unable to cooperate with questionnaires. The cancerous and adjacent normal tissue samples were collected during operation in strict accordance with the specimen collection specifications.

### Cell Culture and Transfection

RC lines (Caki-1, A498, 786-O, and 769-P) and human normal kidney cell line HK-2 were purchased from American Type Culture Collection (Rockville, MD, USA) and cultured in Dulbecco's Modified Eagle Medium (Gibco, Carlsbad, CA, USA) containing 10% fetal bovine serum (FBS; Gibco) and 1% antibiotic (100 U/mL penicillin and 100 mg/mL streptomycin). LncRNA *IRAIN* expression in the cell lines was determined by reverse-transcription quantitative polymerase chain reaction (RT-qPCR) assay. After the cells reached the logarithmic growth phase, the concentration was adjusted to 1 × 10^5^ cells/mL and then the cells were seeded into a 6-well plate containing slides for 24 h. Based on the manufacturer's protocol for Lipofectamine 2000 (Invitrogen, Carlsbad, CA, USA), 75% confluent cells were transfected with 50 ng/mL of pcDNA3.1 [overexpression (oe)-negative control (NC)], pcDNA-lncRNA *IRAIN* (oe-lncRNA *IRAIN*), pcDNA-VEGFA (oe-VEGFA), pcDNA-IRAIN + pcDNA-VEGFA, sh-NC, sh-IRAIN, sh-VEGFA, or sh-IRAIN + sh-VEGFA. All plasmids were constructed by Shanghai GenePharma Co., Ltd. (Shanghai, China).

### RT-qPCR

TRIzol reagent (Invitrogen) was used for isolation of total RNA. cDNA was synthesized according to protocols from the PrimeScript RT reagent Kit (RR047A, Takara, Tokyo, Japan). RT-qPCR was carried out according to the instructions of Fast SYBR Green PCR kit (ABI, Foster City, CA, USA) using an ABI 7500 RT-qPCR system (ABI). The relative expression of target genes was calculated by 2^−ΔΔ*Ct*^ method normalized to that of glyceraldehyde-3-phosphate dehydrogenase (*GAPDH*). The primer sequences are listed in Table 1.

**Table 1:** Primer sequences for RT-qPCR.

**Gene**	**Primer sequence**
LncRNA IRAIN	F: 5′-CGACACATGGTCCAAATCACTGTT-3′
	R: 5′-AGACTCCCCTAGGACTGCCATCT-3′
GAPDH	F: 5′-CGGAGTCAACGGATTTGGTCGTAT-3′
	R: 5′-AGCCTTCTCCATGGTGGTGAAGAC-3′
U6	F: 5′-ACCACAGTCCAT GCCATCAC-3′
	R: 5′-TCCACCACCCTGTTGCTGTA-3′

*RT-qPCR, reverse transcription quantitative polymerase chain reaction; LncRNA IRAIN, long non-coding RNA IRAIN; GAPDH, glyceraldehyde-3-phosphate dehydrogenase; F, forward; R, reverse*.

### Fractionation of Nuclear and Cytoplasmic RNA

The nucleus and cytoplasm were separated using a protein and RNA extraction system kit (Cat. No.: AM1556, Thermo Fisher Scientific, Waltham, MA, USA). The cells were washed 2 times with phosphate-buffered saline (PBS), resuspended in precooled cell fractionation buffer (300 μL), and incubated on ice for 10 min. Following centrifugation at 500 × g for 5 min at 4°C, the supernatant containing the cytoplasmic fraction was collected, leaving behind the nucleus-rich pellet. Nuclear and cytoplasmic RNA was then precipitated after elution for subsequent RT. LncRNA IRAIN expression was identified by RT-qPCR. U6 and GAPDH served as control transcripts for nuclear and cytoplasmic RNA, respectively.

### Western Blot Analysis

Cells were collected by trypsinization and lysed with enhanced radio-immunoprecipitation assay (RIPA) lysis buffer containing protease inhibitors (Boster Biological Technology Co., Ltd., Wuhan, Hubei, China). The protein concentration was determined using a bicinchoninic acid assay kit (Boster). Protein samples were boiled and separated by 10% sodium dodecyl sulfate-polyacrylamide gel electrophoresis and transferred to a polyvinylidene fluoride membrane, followed by 5% bovine serum albumin incubation for 2 h at room temperature. Afterward, the membrane was probed with primary anti-rabbit antibodies to VEGFA (ab52917, 1: 5,000), bcl-2 associated X protein (Bax; ab32503, 1: 5,000), cleaved-caspase3 (ab13847, 1: 500), B-cell lymphoma/leukemia-2 (bcl-2; ab32124, 1: 1,000), E-cadherin (ab40772, 1: 10,000), N-cadherin (ab18203, 1: 1,000), and GAPDH (ab18602, 1: 5,000) overnight at 4°C. On the next day, the membrane was incubated with horseradish peroxidase-labeled secondary goat anti-rabbit antibody to immunoglobulin G (IgG) (ab205718, 1: 2,000) or rabbit anti-mouse antibody to IgG (ab6728, 1: 1,000) at room temperature for 1 h. All antibodies were purchased from Abcam (Cambridge, UK). The membrane was imaged by enhanced chemiluminescence (Millipore, Billerica, MA, USA). The image was subjected to gray value analysis using ImageJ software (Thermo Fisher Scientific). GAPDH was used as the sample loading control.

### Cell Counting Kit-8 (CCK-8) Assay

Cells were cultured in 96-well plates at a density of 1 × 10^3^ cells/well in 100 μL of 10% FBS-containing medium. After 1–5 days of culturing, cell number was measured using a CCK-8 kit (Dojindo, Kumamoto, Japan) according to the manufacturer's instructions. Briefly, 10 μL CCK-8 solution was added to each well for 1 h; a microplate reader was used, and absorbance was read at 450 nm.

### Transwell Assay

Following 12 h culture in a serum-free medium, cells were harvested and resuspended in a serum-free medium (1 × 10^5^ cells/mL). The medium containing 10% FBS was supplemented to the basolateral chamber. A total of 100 μL of the cell suspension was supplemented to the Transwell chamber and incubated for 24 h at 37°C. Cells were fixed with 100% formaldehyde and stained with 1% toluidine blue (Sigma, St. Louis, MO, USA). The stained cells were observed under an inverted light microscope (Carl Zeiss Meditec, Jena, Germany), where five fields were randomly selected for cell counting.

### Flow Cytometry

Cells were collected by centrifugation at 2,000 rpm for 5 min and resuspended in 400 μL of 1 × binding buffer. A total of 5 μL of fluorescein isothiocyanate-conjugated Annexin V was added to the cell suspension, followed by a 15-min incubation in the dark at 4°C. Next, the cells were incubated for 5 min at 4°C in the dark after addition of 10 μL propidium iodide. Cell apoptosis was analyzed on a FACSCalibur flow cytometer within 1 h (BD Biosciences, Franklin Lakes, NJ, USA).

### Methylation-Specific PCR (MSP)

DNA Methylation-Gold™ was applied to detect the methylation status of the *VEGFA* promoter. The methylation reaction primer sequences for MSP amplification were *VEGFA*-MD (5′-TGGGTAGGTAGGTAGGTAGTGGAC-3′) and *VEGFA*-M (5′-ACCTAACAAAACTAAAAATCACGAA-3′). The primer sequences for the unmethylation reaction were *VEGFA*-UD (5′-GGGTAGGTAGGTAGGTAGTGGATGT-3′) and *VEGFA*-UR (5′-ACCTAACAAAACTAAAAATCACAAA-3′). The purified DNA was added to the CT conversion reagent for denaturation and hydrogen sulfate transformation. The reaction column was used to desulfurize and purify DNA that was subjected to subsequent PCR. PCR conditions were as follows: pre-denaturation at 95°C for 10 min, 35 cycles of denaturation at 95°C for 45 s, 56°C (methylation)/45°C (unmethylation) for 45 s, and annealing at 72°C for 45 s, with the last extension at 72°C for 10 min. The reaction product was subjected to agarose gel electrophoresis, followed by image analysis by a gel electrophoresis imaging system.

### RNA Immunoprecipitation (RIP)

The direct interaction of lncRNA *IRAIN* with methyltransferases (Dnmt1, Dnmt3a, Dnmt3b) was determined using a RIP kit (Millipore). Cells were lysed, and the supernatant was collected following 10 min of centrifugation at 4°C. A portion of the cell extract was used as input, and the rest was immunoprecipitated with the indicated antibody. Briefly, in each reaction, 50-μL magnetic beads were resuspended in 100 μL RIP wash buffer and 5 μg of antibody was added to the beads. The magnetic bead–antibody complex was resuspended in 900 μL RIP wash buffer, followed by overnight incubation with 100 μL of cell extract at 4°C. Samples were placed on a magnetic base to collect the magnetic bead–protein complex and then treated with proteinase K to extract RNA for downstream analysis of lncRNA *IRAIN* expression by RT-qPCR. Antibodies used in RIP included anti-Dnmt1 (1: 100, ab13537), anti-Dnmt3a (1: 100, ab2850), and anti-Dnmt3b (1:50, ab2851) (being mixed at room temperature for 30 min). Rabbit anti-human IgG (1: 100, ab109489) served as a NC. All antibodies were obtained from Abcam.

### RNA-Protein Pull-Down

RC cells were transfected with wild-type (WT) biotinylated lncRNA *IRAIN* (50 nM) and mutant-type (MUT) biotinylated lncRNA *IRAIN* (50 nM). Cells were harvested and vortexed after 48 h of incubation. Specific cell lysis buffer (Ambion, Austin, Texas, USA) was added to the cells followed by 10 min of incubation on ice. The 3-h lysate incubation was performed with M-280 streptavidin magnetic beads (Sigma) pre-coated with RNase-free and yeast tRNA (Sigma) at 4°C. Cells were washed twice with cold lysis buffer, 3 times with low salt buffer, and once with high salt buffer. Total protein was extracted with a high-efficiency RIPA buffer. Dnmt1, Dnmt3a, and Dnmt3b expression was analyzed by Western blot analysis.

### Chromatin Immunoprecipitation (ChIP)

After cross-linking with formalin (1% of final concentration), cells were sonicated and incubated with anti-Dnmt1 (1: 100, ab13537), anti-Dnmt3a (1: 100, ab2850), and anti-Dnmt3b (1: 50, ab2851) antibodies (Abcam). Then, protein agarose/salmon sperm DNA was added to the sample and bead complexes were washed to remove non-specific binding molecules. The enriched precipitated complex was eluted and de-cross-linked. The purified DNA was subjected to qPCR.

### Tumor Xenografts in Nude Mice

Forty-five specific pathogen-free grade female nude mice (aged 5 weeks, weighing 18–20 g) were purchased from the Animal Center Laboratory of Henan University. The 786-O RC cells were transfected with oe-NC and oe-*IRAIN* alone or in the presence of oe-*VEGFA*. Transfected cells (6 × 10^6^ cells) were resuspended and mixed with Matrigel at a volume ratio of 1: 1. A total of 3 × 10^6^ cells (0.2 mL of suspension) were inoculated subcutaneously into the right axilla of the mice in order to generate xenografts (*n* = 15). The tumor growth was observed, and tumor volume and weight were determined.

### Statistical Analysis

The data were realized using SPSS 21.0 statistical software (IBM Corp. Armonk, NY, USA). Measurement data were shown as mean ± standard deviation. Normal distributed unpaired data with equal variance between two groups were analyzed using unpaired *t*-test. Comparisons among multiple groups were conducted by one-way analysis of variance (ANOVA) with Tukey's *post-hoc* test, and data at different time points were processed by repeated-measures ANOVA, followed by Bonferroni *post-hoc* test. A value of *p* < 0.05 indicated significant difference.

## Results

### LncRNA *IRAIN* Is Poorly Expressed in RC Tissues and Cells

**Figure 1:**
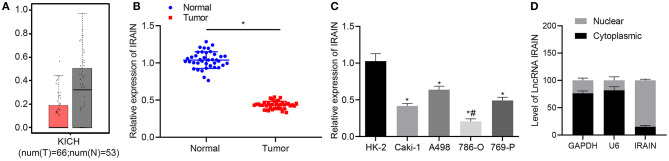
Lncrna *IRAIN* expression is low in RC tissues and cells. **(A)** The expression of lncRNA *IRAIN* based on the ENSG00000259424 profile; the x-axis indicates the sample type, and the y-axis indicates the expression of lncRNA *IRAIN*. **(B)** The expression of lncRNA *IRAIN* in RC tissues and normal adjacent tissues measured by RT-qPCR (*n* = 40) normalized to GAPDH. **(C)** The expression of lncRNA *IRAIN* in human renal cell line (HK-2) and RC cell lines (Caki-1, A498, 786-O, and 769-P) measured by RT-qPCR normalized to *GAPDH*. **(D)** Nuclear and cytoplasmic expression of lncRNA *IRAIN* in HK-2 cells determined by RT-qPCR. **(B)** *p < 0.05 tumor tissues *vs*. adjacent normal tissues. **(C)** *p < 0.05 786-O, Caki-1, A498, or 769-P cell line *vs*. HK-2 cell line. ^#^*p* < 0.05 786-O cell line *vs*. Caki-1, A498, or 769-P cell line. Measurement data were shown as mean ± standard deviation. Data between two groups were analyzed using unpaired *t*-test. Comparisons among multiple groups were conducted by one-way ANOVA with Tukey's *post-hoc* test. The cell experiment was repeated independently 3 times.

The expression profile ENSG00000259424 was employed to predict the expression of lncRNA *IRAIN* in RC, which showed low expression of lncRNA *IRAIN* in RC (Figure 1A). Next, we measured lncRNA *IRAIN* expression in resected RC tissues and adjacent normal tissues from 42 confirmed RC patients. RT-qPCR revealed downregulation of the lncRNA *IRAIN* in RC tissues compared to adjacent normal tissues (*p* < 0.05; Figure 1B). These results are listed in Table 2, indicating that lncRNA *IRAIN* expression was not significantly correlated with gender and age but displayed a significant correlation with tumor grade, TNM stage, and lymph node metastasis (*p* < 0.05). Next, we measured the lncRNA *IRAIN* expression in the human renal cell line (HK-2) and four RC cell lines (Caki-1, A498, 786-O, and 769-P). The results showed that in comparison with HK-2 cells, lncRNA *IRAIN* was poorly expressed in Caki-1, A498, 786-O, and 769-P cells (*p* < 0.05; Figure 1C). To better understand the function of the lncRNA *IRAIN*, we investigated its subcellular localization in the nucleus vs. cytoplasm. Our results revealed that the lncRNA *IRAIN* was localized primarily in the nucleus (Figure 1D). The above data show that lncRNA *IRAIN* is expressed at low levels in RC tissues and cell lines compared to healthy renal tissues.

**Table 2:** Correlation between lncRNA IRAIN expression and clinicopathological features of patients with RC.

**Clinicopathological features**	**Cases (n)**	**Expression of lncRNA IRAIN**	***p***
Age (years)			0.057
<60	23	0.423 ± 0.051	
≥ 60	17	0.444 ± 0.046	
Gender			0.188
Male	22	0.439 ± 0.051	
Female	18	0.424 ± 0.050	
Tumor grade			0.001
Grades I–II	28	0.458 ± 0.035	
Grade III	12	0.370 ± 0.019	
TNM stage			0.001
Early stage	30	0.454 ± 0.038	
Mid and late stages	10	0.367 ± 0.019	
Lymph node metastasis			0.001
No	19	0.472 ± 0.034	
Yes	21	0.396 ± 0.033	

*lncRNA IRAIN, long non-coding RNA IRAIN; RC, renal carcinoma; TNM, tumor-node metastasis*.

### Overexpression of LncRNA *IRAIN* Inhibits the Proliferation and Migration of RC Cells While Promoting Their Apoptosis

**Figure 2:**
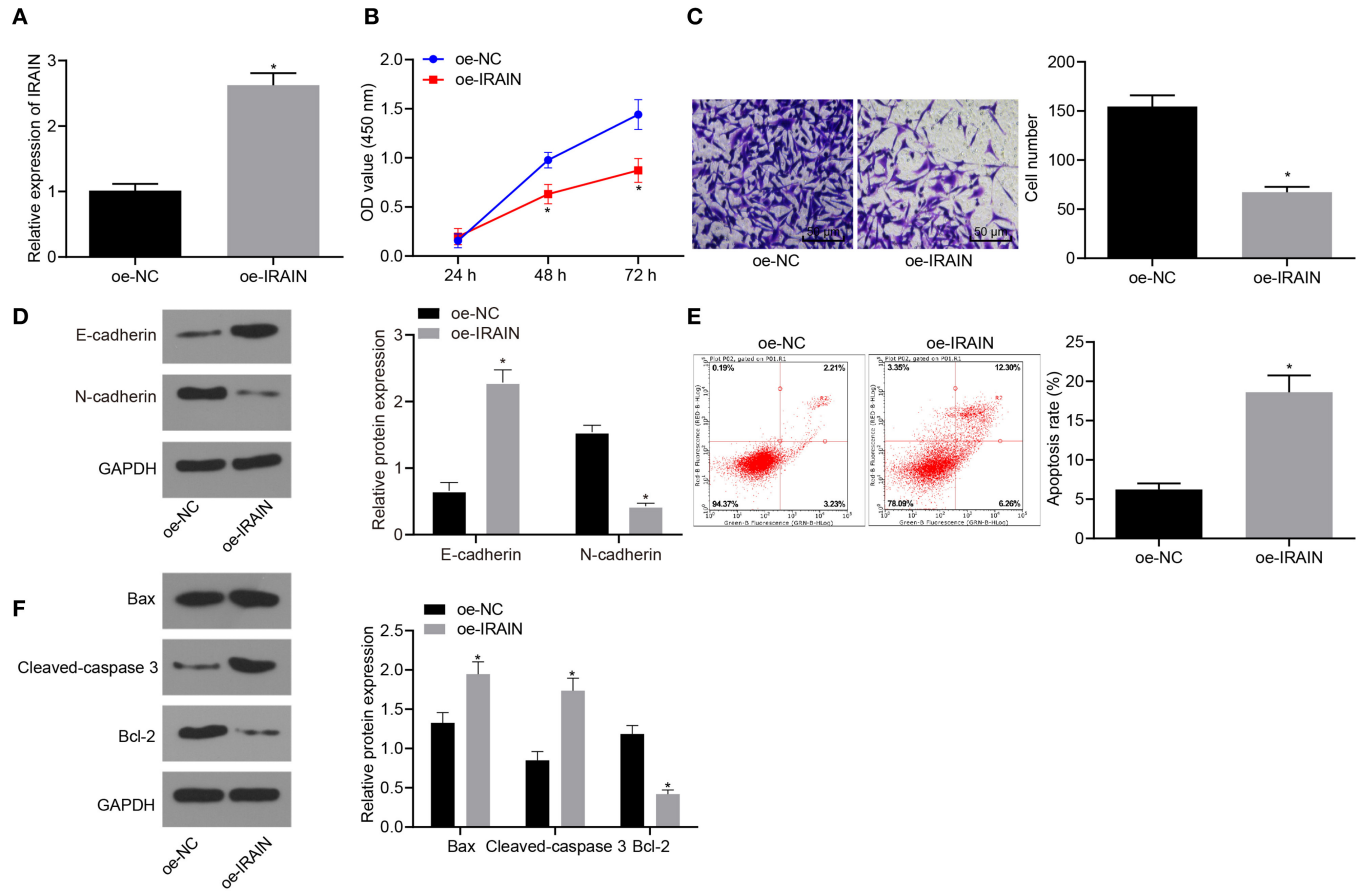
Upregulation of lncRNA *IRAIN* results in a decrease in proliferation and migration and an increase in apoptosis in RC cells. The 786-O cells were co-transfected with oe-*IRAIN* or oe-NC. **(A)** The expression of lncRNA *IRAIN* in 786-O cells examined by RT-qPCR normalized to *GAPDH*. **(B)** 786-O cell proliferation detected by CCK-8 assay. **(C)** 786-O cell migration assessed by a Transwell assay (×200). **(D)** Protein levels of EMT-related factors (E-cadherin and N-cadherin) in 786-O cells normalized to GAPDH determined by Western blot analysis. **(E)** 786-O cell apoptosis measured by flow cytometry. **(F)** The expression of apoptosis-related factors (bcl-2, Bax, and cleaved-caspase3) analyzed by Western blot analysis normalized to GAPDH. *p < 0.05 786-O cells transfected with oe-IRAIN *vs*. 786-O cells transfected with oe-NC. Measurement data were shown as mean ± standard deviation. Data between two groups were compared using unpaired *t*-test. Data at different time points were compared by repeated-measures ANOVA, followed by Bonferroni *post-hoc* test. The experiment was repeated independently 3 times.

To elucidate the effect of lncRNA *IRAIN* on proliferation, migration and apoptosis of RC cells, cell proliferation, and migration and apoptosis after overexpression of lncRNA *IRAIN* were studied in 786-O cells, first, RT-qPCR was used to measure lncRNA *IRAIN* expression, revealing significantly increased levels of lncRNA *IRAIN* in 786-O cells transfected with oe-*IRAIN* (Figure 2A). Next, CCK-8 (Figure 2B), Transwell (Figure 2C), Western blot (Figure 2D), and flow cytometric (Figure 2E) assays were performed to study cell proliferation, migration, and apoptosis. The cell proliferation and migration of 786-O cells overexpressing lncRNA *IRAIN* were markedly reduced, and apoptosis was increased, which was accompanied by upregulation of E-cadherin and downregulation of N-cadherin. Western blot analysis showed that Bax and cleaved-caspase3 expression was markedly increased but bcl-2 expression was notably decreased in 786-O cells transfected with oe-*IRAIN* (Figure 2F). In conclusion, overexpression of lncRNA *IRAIN* restrained cell proliferation and migration while inducing apoptosis in RC cell lines.

### VEGFA Is Highly Expressed in RC Tissues and Cells

**Figure 3:**
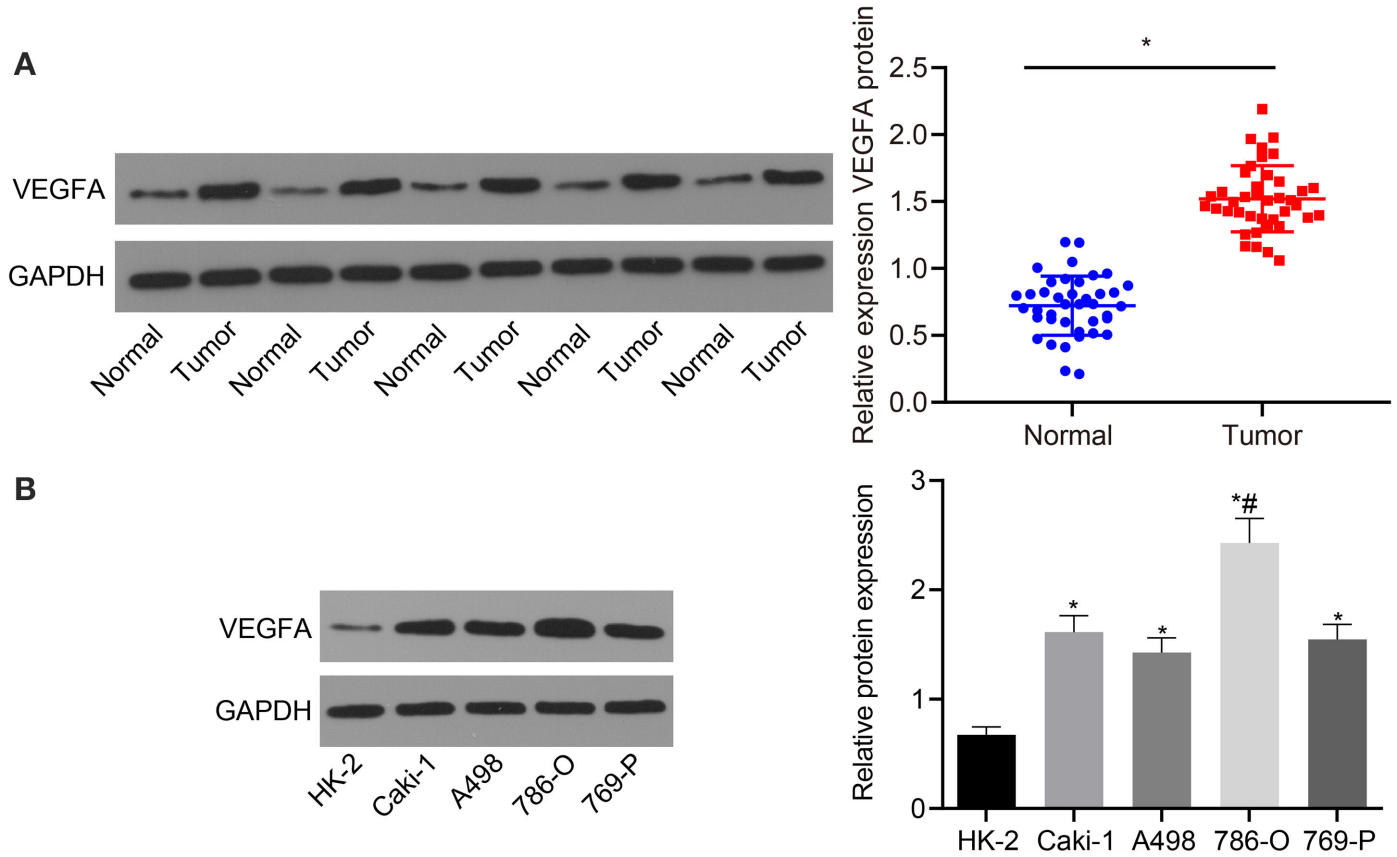
VEGFA is upregulated in RC tissues and cells. **(A)** The expression of VEGFA in RC tissues and adjacent normal tissues determined by Western blot analysis normalized to GAPDH (*n* = 40). **(B)** The expression of VEGFA in a human normal renal cell line (HK-2) and RC cell lines (Caki-1, A498, 786-O, and 769-P) measured by Western blot analysis normalized to GAPDH. **(A)** *p < 0.05 tumor tissues *vs*. adjacent normal tissues. **(B)** *p < 0.05 786-O, Caki-1, A498, or 769-P cell line *vs*. HK-2 cell line. ^#^*p* < 0.05 786-O cell line *vs*. Caki-1, A498, or 769-P cell line. Measurement data were expressed as mean ± standard deviation. Data between two groups were compared using an unpaired *t*-test. Comparisons among multiple groups were conducted by one-way ANOVA with Tukey's *post-hoc* test. The experiment was repeated independently 3 times.

Next, VEGFA expression was assessed in RC tissues and adjacent normal tissues. As shown in Figure 3A, VEGFA expression was significantly increased in RC tissues compared with adjacent normal tissues (*p* < 0.05). VEGFA expression was also assessed in the normal human renal cell line (HK-2) as well as RC cell lines (Caki-1, A498, 786-O, and 769-P). The results suggest that in contrast to HK-2 cells, VEGFA was highly expressed in the Caki-1, A498, 786-O, and 769-P cell lines, with the highest expression in the 786-O cell line (*p* < 0.05; Figure 3B). Thus, we determined that VEGFA is highly expressed in RC patient samples and cell lines compared to healthy tissues.

### *VEGFA* Downregulation Inhibits Proliferation and Migration While Increasing Apoptosis of RC Cells

**Figure 4:**
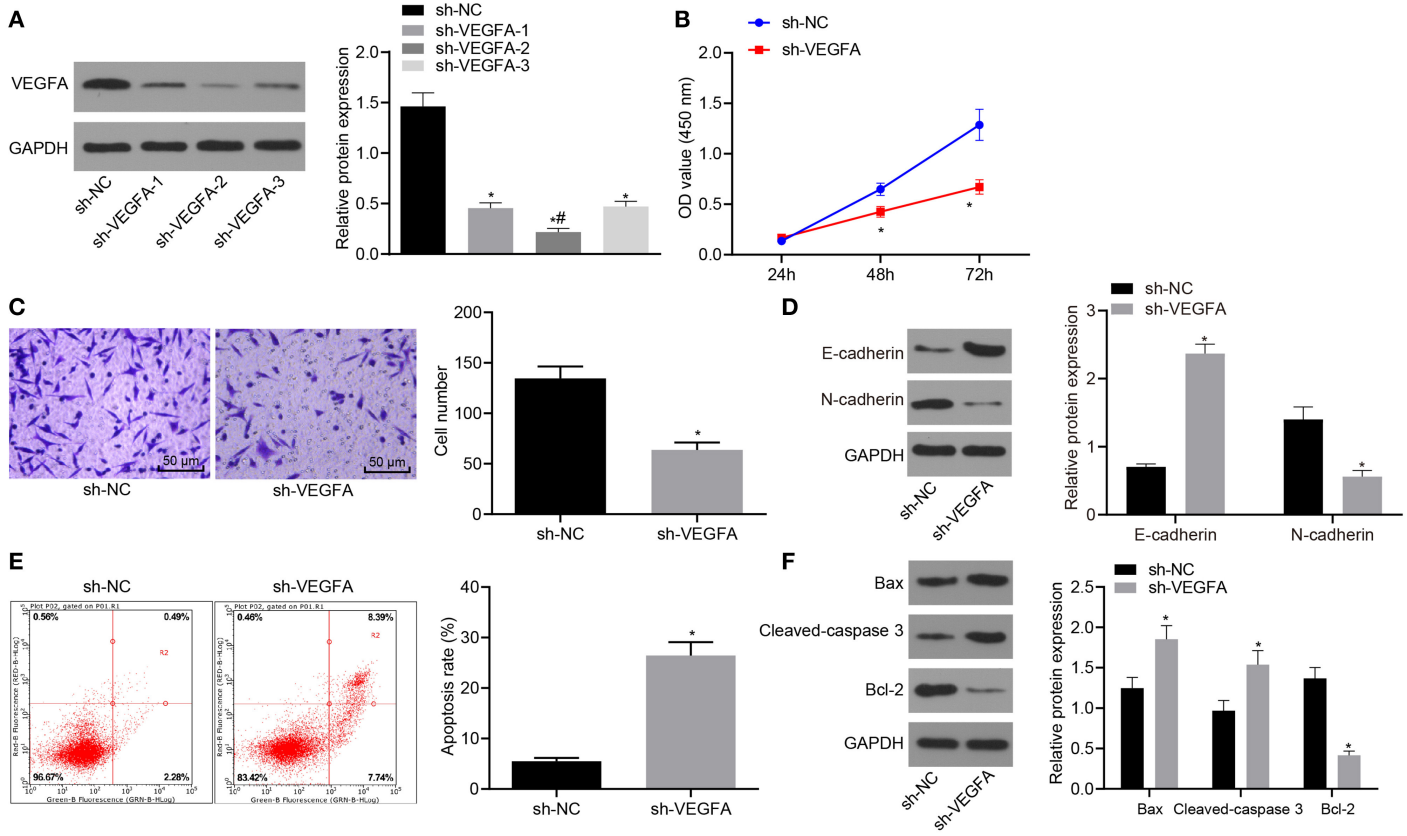
Downregulation of VEGFA suppresses RC cell proliferation and migration and accelerates cell apoptosis. **(A)** The silencing efficiency of VEGFA in 786-O cells as examined by Western blot analysis normalized to GAPDH. The 786-O cells were transfected with sh-NC or sh-*VEGFA*. **(B)** 786-O cell viability assessed by CCK-8 assay. **(C)** 786-O cell migration examined by the Transwell assay (×200). **(D)** Protein levels of EMT-related factors (E-cadherin and N-cadherin) in 786-O cells normalized to GAPDH determined by Western blot analysis. **(E)** 786-O cell apoptosis analyzed by flow cytometry. **(F)** The expression of apoptosis-related factors (bcl-2, Bax, and cleaved-caspase3) in 786-O cells determined by Western blot analysis normalized to GAPDH. *p < 0.05 786-O cells transfected with sh-*VEGFA vs*. 786-O cells transfected with sh-NC. ^#^*p* < 0.05 786-O cells transfected with sh-*VEGFA*-2 *vs*. 786-O cells transfected with sh-*VEGFA*-1 or sh-*VEGFA*-3. Measurement data were expressed as mean ± standard deviation. Data between two groups were compared using unpaired *t*-test. Data at different time points were compared by repeated-measures ANOVA, followed by Bonferroni *post-hoc* test. The experiment was repeated independently 3 times.

After *VEGFA* was knocked down in the 786-O RC cell line, we characterized cell proliferation, migration, and apoptosis. Three putative silencing sh-RNAs were designed and transfected into 786-O cells. Silencing efficiency was measured by Western blot analysis. The data showed that compared with cells transfected with the sh-NC, VEGFA expression was strikingly diminished in cells transfected with all sh-RNAs but especially in cells transfected with sh-*VEGFA*-2. Therefore, we selected sh-*VEGFA*-2 for all subsequent experiments (Figure 4A). The cell viability, migration, and apoptosis of the 786-O RC cell line were examined by CCK-8 assay (Figure 4B), Transwell assay (Figure 4C), Western blot analysis (Figure 4D), and flow cytometry (Figure 4E). Proliferation and migration of 786-O cells treated with sh-*VEGFA* were considerably reduced, and cell apoptosis was elevated characterized by upregulation of E-cadherin and downregulation of N-cadherin. Western blot analysis further displayed that Bax and cleaved-caspase3 expression was significantly enhanced, accompanied by a reduction in bcl-2 expression in sh-*VEGFA*-transfected 786-O cells (Figure 4F). Taken together, our data showed that *VEGFA* silencing inhibited cell proliferation and migration while promoting the apoptosis of the 786-O RC cell line.

### LncRNA *IRAIN* Regulates *VEGFA* Expression by Recruiting DNA Methylases to the *VEGFA* Promoter Region

**Figure 5:**
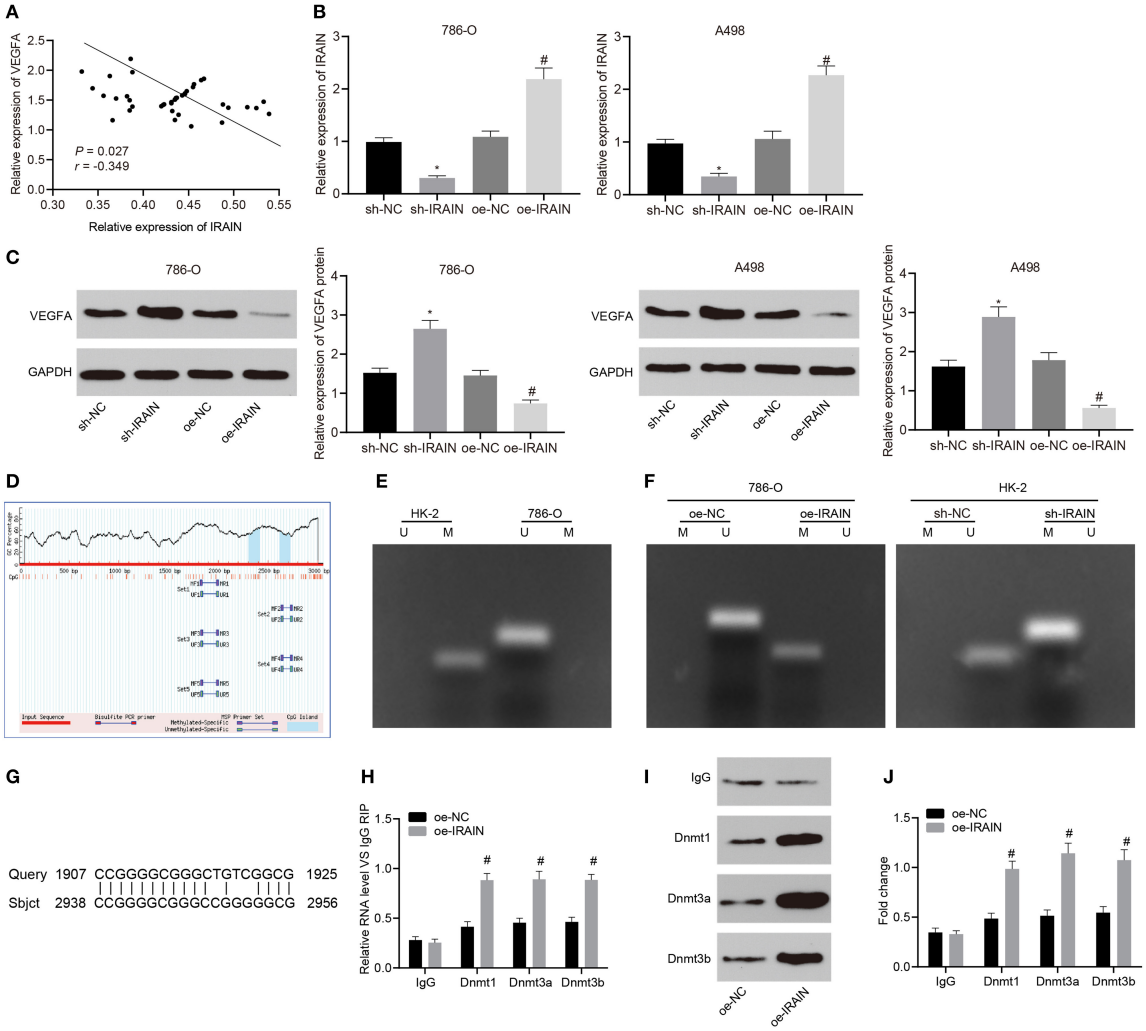
Upregulation of lncRNA *IRAIN* downregulates VEGFA *via* recruitment of DNA methylase to the promoter region of VEGFA. **(A)** The correlation analysis of the expression between lncRNA *IRAIN* and VEGFA. **(B)** The expression of lncRNA *IRAIN* in 786-O and A498 cells after alteration of lncRNA *IRAIN* measured by RT-qPCR normalized to GAPDH. **(C)** The expression of VEGFA in 786-O and A498 cells after alteration of lncRNA *IRAIN* measured by Western blot analysis normalized to GAPDH. **(D)** CpG islands in the *VEGFA* promoter region predicted on MethPrimer (http://www.urogene.org/cgi-bin/methprimer/methprimer.cgi). **(E)** Methylation status at specific sites in HK-2 and 786-O cells detected by MSP. **(F)** Methylation status at specific sites in HK-2 and 786-O cells in response to oe-*IRAIN* detected by MSP. **(G)** Binding form between the lncRNA *IRAIN* and VEGFA promoter regions examined by a Blast online alignment. **(H)** The binding of lncRNA *IRAIN* and methyltransferase assessed by RIP assay. **(I)** The binding of lncRNA *IRAIN* and methyltransferase assessed by RNA-protein pull down. **(J)** Methyltransferase enrichment in the VEGFA promoter region detected by ChIP assay. *p < 0.05 cells transfected with sh-*IRAIN vs*. cells transfected with sh-NC. ^#^*p* < 0.05 cells transfected with oe-*IRAIN vs*. cells transfected with oe-NC. Measurement data were expressed as mean ± standard deviation. Data between two groups were compared using unpaired *t*-test. The experiment was repeated independently 3 times.

Since our results had revealed the functional role of lncRNA *IRAIN* and *VEGFA* in RC, we next explored whether lncRNA *IRAIN* can regulate *VEGFA* expression. A correlation analysis revealed that the lncRNA *IRAIN* negatively correlated with *VEGFA* expression (*p* < 0.05; Figure 5A). LncRNA *IRAIN* expression was analyzed in A498 and 786-O cells by RT-qPCR. The results showed that lncRNA *IRAIN* expression was potently significantly reduced in A498 and 786-O cells transfected with sh-*IRAIN* but notably increased in those treated with oe-*IRAIN* (Figure 5B). Furthermore, Western blot analysis confirmed these results as VEGFA expression in sh-*IRAIN*-transfected A498 and 786-O cells was significantly increased, while oe-*IRAIN*-transfected A498 and 786-O cells had significantly decreased VEGFA levels (Figure 5C). This data indicated that lncRNA *IRAIN* could potentially play a role in downregulating VEGFA expression.

To explore this possibility, we analyzed the *VEGFA* promoter region using MethPrimer and found one CpG island (Figure 5D). MSP was applied to detect the methylation status of this specific site in the *VEGFA* promoter region of HK-2 and 786-O cells. Our results showed that there was methylation at this specific site in the *VEGFA* promoter region of HK-2 cells but no methylation was present at the same site in the *VEGFA* promoter of 786-O cells (Figure 5E). Next, we performed MSP after overexpressing the lncRNA *IRAIN*. No methylation was observed in 786-O cells treated with oe-NC; however, methylation was present in 786-O cells treated with oe-*IRAIN*. At the same time, the lncRNA *IRAIN* was knocked down in HK-2 cells and MSP was performed, which also revealed significant methylation in HK-2 cells transfected with sh-NC while methylation was absent in HK-2 cells transfected with sh-*IRAIN* (Figure 5F). Using blast alignment revealed that lncRNA *IRAIN* bound to *VEGFA* promoters in an RNA-DNA hybrid form (Figure 5G). RIP assay was used next in order to analyze the interaction between lncRNA *IRAIN* and DNA methyltransferase. The results showed markedly enhanced binding of Dnmt1, Dnmt3a, and Dnmt3b to the VEGFA promoter in 786-O cells overexpressing the lncRNA *IRAIN* (Figure 5H). Furthermore, an RNA-protein pull-down assay showed that Bio-*IRAIN*-WT was able to pull down Dnmt1, Dnmt3a, and Dnmt3b, but the Bio-*IRAIN*-MUT construct was not able pull down these proteins (Figure 5I). Next, ChIP assay demonstrated a significant increase in the enrichment of Dnmt1, Dnmt3a, and Dnmt3b in the *VEGFA* promoter region after overexpressing the lncRNA *IRAIN* (Figure 5J). In summary, upregulation of the lncRNA *IRAIN* decreased VEGFA expression by recruiting DNA methylases to the promoter region of *VEGFA*.

### By Inactivating VEGFA, LncRNA *IRAIN* Inhibits Proliferation and Migration, While Increasing the Apoptosis of RC Cells

**Figure 6:**
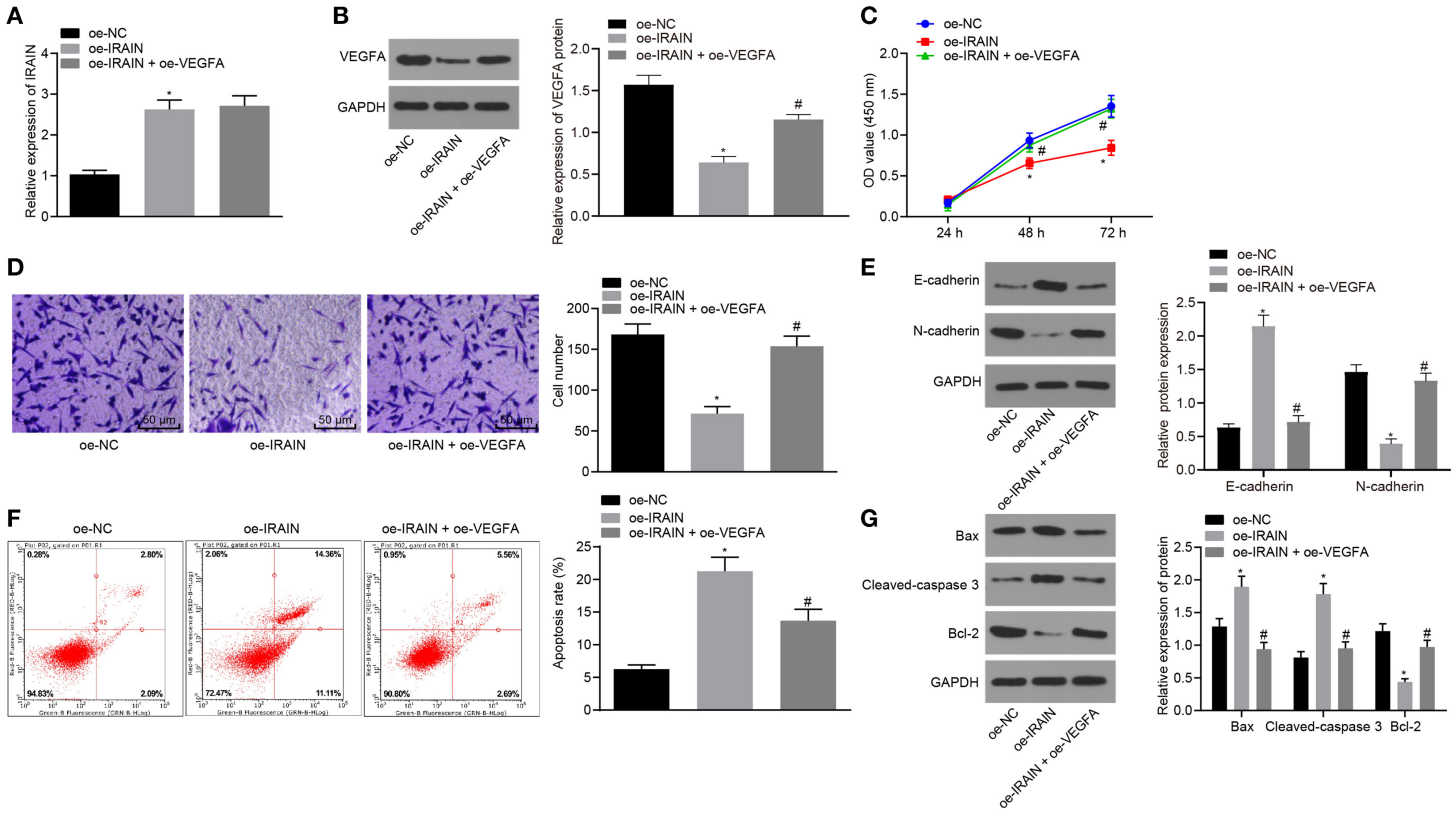
Overexpression of lncRNA *IRAIN* restrains 786-O cell proliferation and migration and accelerates 786-O cell apoptosis through inhibition of VEGFA. The 786-O cells were transfected with oe-NC, oe-*IRAIN*, or oe-*IRAIN* + oe-*VEGFA*. **(A)** The expression of lncRNA *IRAIN* in 786-O cells examined by RT-qPCR normalized to *GAPDH*. **(B)** The expression of VEGFA in 786-O cells determined by Western blot analysis normalized to GAPDH. **(C)** Proliferation of 786-O cells measured by CCK-8 assay. **(D)** Migration of 786-O cells examined by Transwell assay (×200). **(E)** Protein levels of EMT-related factors (E-cadherin and N-cadherin) in 786-O cells normalized to GAPDH determined by Western blot analysis. **(F)** Apoptosis of 786-O cells detected by flow cytometry. **(G)** The expression of apoptosis-related factors measured by Western blot analysis normalized to GAPDH. *p < 0.05 786-O cells transfected with oe-IRAIN *vs*. 786-O cells transfected with oe-NC. ^#^*p* < 0.05 786-O cells transfected with oe-IRAIN + oe-VEGFA *vs*. 786-O cells transfected with oe-*IRAIN*. Measurement data were expressed as mean ± standard deviation. Comparisons among multiple groups were conducted by one-way ANOVA with Tukey's *post-hoc* test. Data at different time points were compared by repeated-measures ANOVA, followed by Bonferroni *post-hoc* test. The experiment was repeated independently 3 times.

Next, in order to investigate the effects of lncRNA *IRAIN*-regulating VEGFA on the proliferation, migration, and apoptosis of RC cells, we transfected A498 and 786-O cells with oe-*IRAIN* alone or in the presence of oe-*VEGFA*. LncRNA *IRAIN* expression was measured by RT-qPCR, which showed that lncRNA *IRAIN* expression was significantly increased in lncRNA *IRAIN*-overexpressing A498 and 786-O cells. There was no significant difference regarding the expression of lncRNA *IRAIN* between cells transfected with oe-*IRAIN* and cells transfected with oe-*IRAIN* + oe-*VEGFA* (*p* > 0.05; Figure 6A, Supplementary Figure 1A). Western blot analysis demonstrated a significant decrease of VEGFA in oe-*IRAIN*-transfected cells, which was restored by oe-*VEGFA* transfection (Figure 6B, Supplementary Figure 1B).

CCK-8 (Figure 6C, Supplementary Figure 1C), Transwell (Figure 6D, Supplementary Figure 1D), Western blot analysis (Figure 6E, Supplementary Figure 1E), and flow cytometry (Figure 6F, Supplementary Figure 1F) assays indicated that in response to oe-*IRAIN*, 786-O and A498 cell proliferation and migration were notably diminished, while the cell apoptosis was increased, E-cadherin expression was elevated, and N-cadherin expression was diminished. All effects were restored upon overexpressing *VEGFA*. The expression of apoptosis-related factors was measured by Western blot analysis, and, as documented in Figure 6G, Supplementary Figure 1G, Bax and cleaved-caspase3 expression was significantly increased but bcl-2 expression was notably decreased in 786-O and A498 cells in response to oe-*IRAIN*, which was reversed after oe-*VEGFA* treatment. These data show that overexpression of the lncRNA *IRAIN* suppressed the proliferation and migration of RC cells by inhibiting *VEGFA*, while apoptosis is induced.

**Figure 7:**
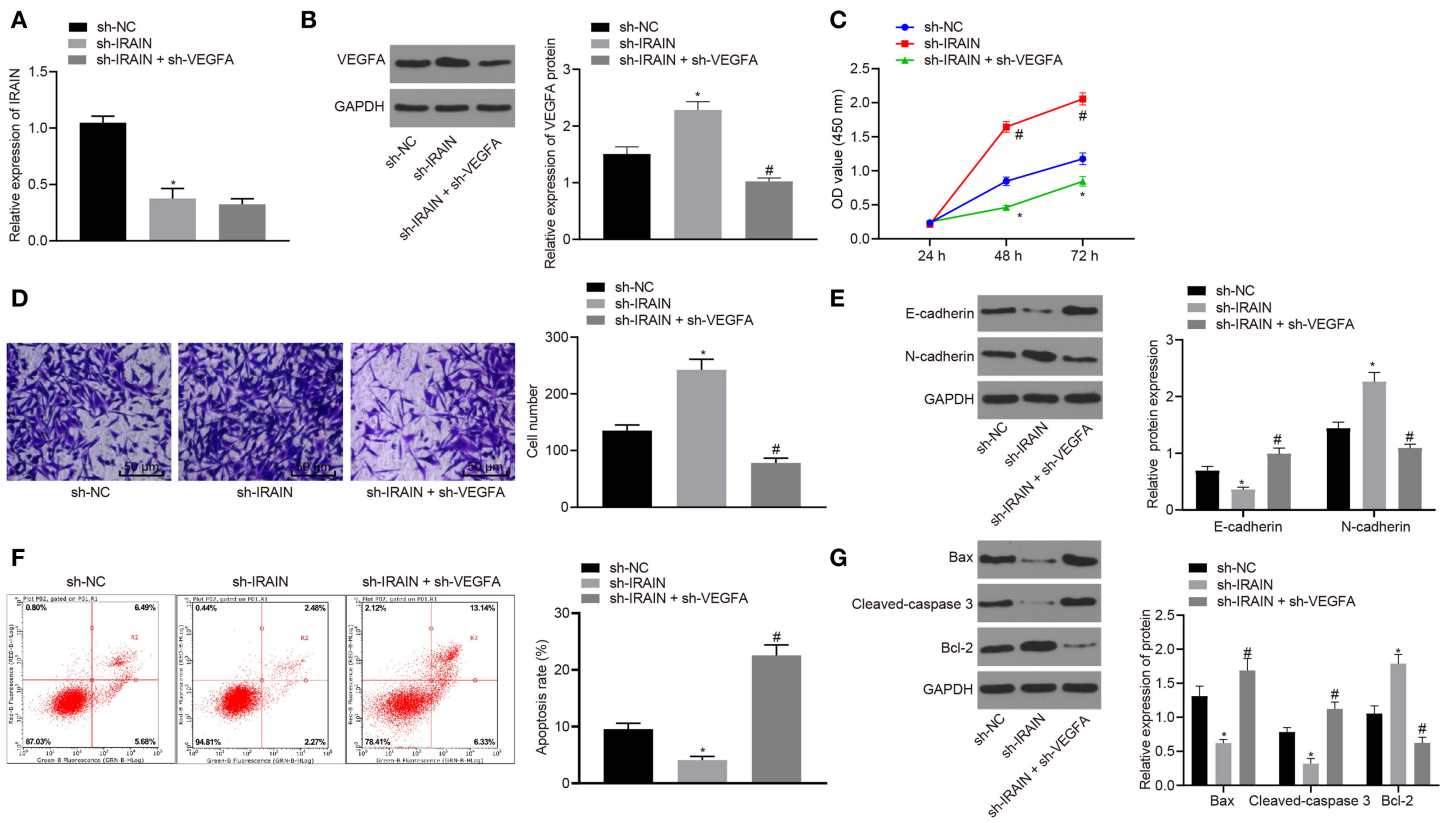
Upregulated lncRNA *IRAIN* suppresses A498 cell proliferation and migration and induces apoptosis through inhibition of VEGFA. The A498 cells were transfected with sh-NC, sh-*IRAIN*, or sh-*IRAIN* + sh-*VEGFA*. **(A)** The expression of the lncRNA *IRAIN* in A498 cells was examined by RT-qPCR and normalized to *GAPDH*. **(B)** The expression of VEGFA in A498 cells determined by Western blot analysis normalized to GAPDH. **(C)** Proliferation of A498 cells measured by CCK-8 assay. **(D)** Migration of A498 cells examined by Transwell assay (×200). **(E)** Protein levels of EMT-related factors (E-cadherin and N-cadherin) in A498 cells normalized to GAPDH determined by Western blot analysis. **(F)** Apoptosis of A498 cells detected by flow cytometry. **(G)** The expression of apoptosis-related factors (bcl-2, Bax, and cleaved-caspase3) measured by Western blot analysis normalized to GAPDH. *p < 0.05 A498 cells transfected with sh-*IRAIN vs*. A498 cells transfected with sh-NC. ^#^*p* < 0.05 A498 cells transfected with sh-*IRAIN* + sh-*VEGFA vs*. A498 cells transfected with sh-*IRAIN*. Measurement data were expressed as mean ± standard deviation. Comparisons among multiple groups were conducted by one-way ANOVA with Tukey's *post-hoc* test. Data at different time points were compared by repeated measures ANOVA, followed by Bonferroni *post-hoc* test. The experiment was repeated independently 3 times.

For further exploration of the regulatory role of lncRNA *IRAIN* on RC cell proliferation, migration, and apoptosis, we knocked down lncRNA *IRAIN* with/without knockdown of *VEGFA* in A498 cells, followed by evaluation of the resulting cell proliferation, migration, and invasive ability. RT-qPCR was performed first to determine the expression of lncRNA *IRAIN* and results showed presence of sh-*IRAIN* corresponded to significantly downregulated lncRNA *IRAIN* (*p* < 0.05) yet the addition of sh-*VEGFA* made lncRNA *IRAIN* expression insignificantly different (*p* > 0.05) (Figure 7A). Further, Western blot analysis for VEGFA quantification revealed that VEGFA expression was significantly diminished by sh-*IRAIN*, the effect of which was abolished by sh-*VEGFA* (*p* < 0.05) (Figure 7B). Subsequently, CCK-8 (Figure 7C), Transwell (Figure 7D), and flow cytometry assays (Figure 7F) were followed to assess A498 cell proliferation, migration, and apoptosis. Meanwhile, Western blot analysis was carried out to quantify EMT-related factors (E-cadherin and N-cadherin) (Figure 7E) and apoptosis-related factors (Bax, cleaved-caspase3, and bcl-2) (Figure 7G). It was found that in A498 cells carrying sh-*IRAIN*, cell proliferation and migration were promoted and cell apoptosis was inhibited, accompanied by lower levels of E-cadherin, Bax, and cleaved-caspase3 as well as higher levels of N-cadherin and bcl-2. Opposite changing tendency was observed in A498 cells carrying sh-*IRAIN* + sh-*VEGFA*. Taken together, these findings indicated that silencing lncRNA *IRAIN* contributed to RC cell proliferation and migration while curbing cell apoptosis by upregulating *VEGFA*.

### LncRNA *IRAIN* Impedes Tumor Growth of RC *via* VEGFA Suppression *in vivo*

**Figure 8:**
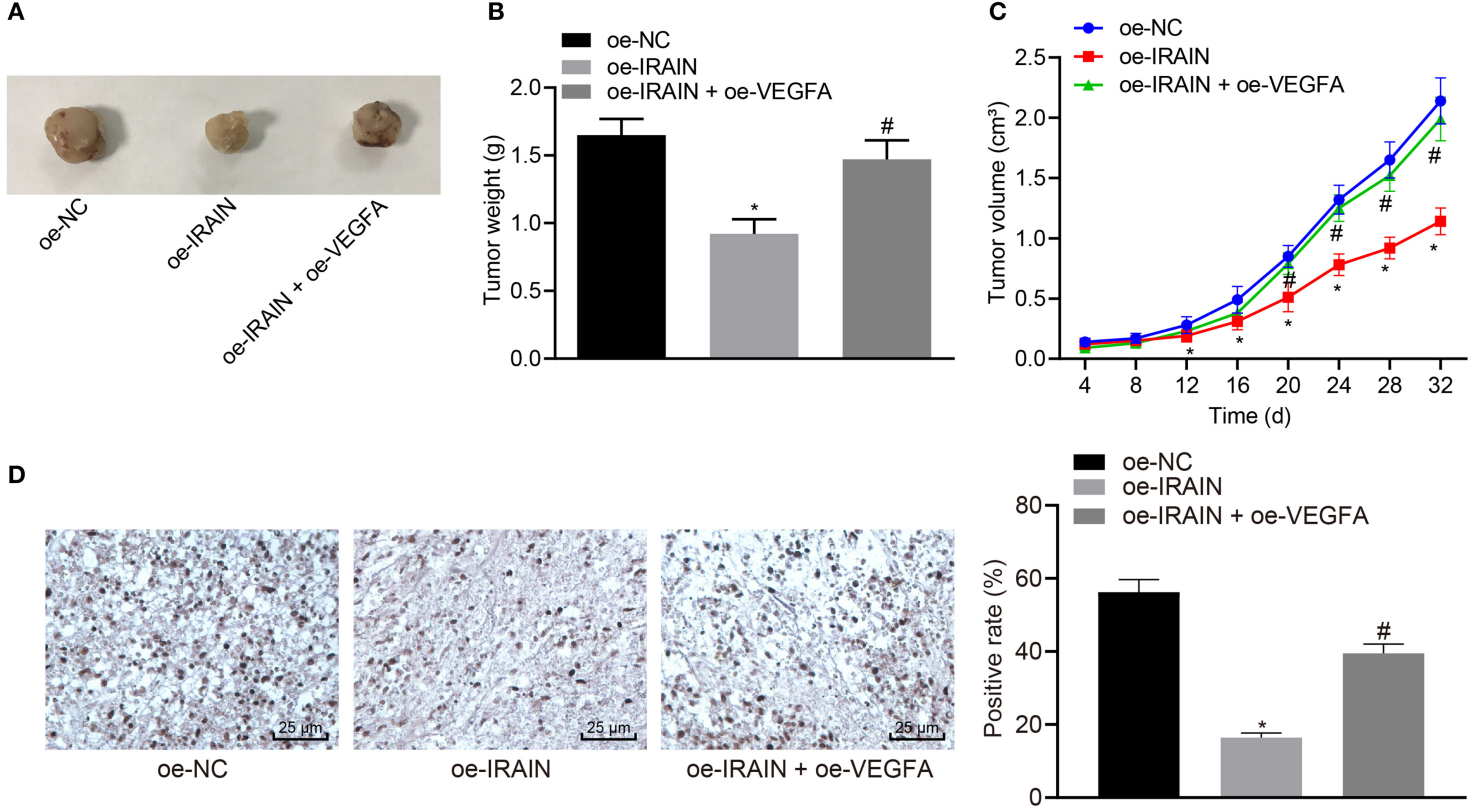
Overexpression of lncRNA *IRAIN* restrains tumor growth of RC *in vivo* through suppression of VEGFA. Mice were injected with 786-O cells which were transfected with oe-NC, oe-*IRAIN*, or oe-*IRAIN* + oe-*VEGFA*. **(A)** Tumor size of nude mice. **(B)** Tumor weight of nude mice. **(C)** The tumor growth curve. **(D)** KI67 expression in tumor tissues identified by immunohistochemistry. *p < 0.05 mice treated with oe-*IRAIN vs*. mice treated with oe-NC. ^#^*p* < 0.05 mice treated with oe-*IRAIN* + oe-*VEGFA vs*. mice treated with oe-*IRA*IN. Measurement data were expressed as mean ± standard deviation. Comparisons among multiple groups were conducted by one-way ANOVA with Tukey's *post-hoc* test. Data at different time points were compared by repeated measures ANOVA, followed by Bonferroni *post-hoc* test. *n* = 15. The experiment was repeated independently 3 times.

Stably transfected 786-O cells were inoculated into nude mice in order to explore the effects of lncRNA *IRAIN* and *VEGFA* on tumor growth of RC *in vivo*. The tumor growth curve, tumor size, and weight of nude mice were analyzed. It became apparent that tumor size and weight were markedly reduced in mice injected with oe-*IRAIN*-transfected cells, which was rescued by treatment with oe-*VEGFA* (Figures 8A–C). Then, KI67 expression was detected by immunohistochemistry, the results of which revealed significantly higher KI67 levels in the presence of oe-*IRAIN*, while further delivery of oe-*VEGFA* counterweighed the effects of oe-*IRAIN* (Figure 8D). In conclusion, upregulated lncRNA *IRAIN* repressed tumor growth of RC through inhibition of *VEGFA in vivo*.

## Discussion

Malignant RC composes 2% of the global cancer burden, with increasing incidence rate (2). Thus, RCC tumor biology is urgently needed to understand the progress and to identify novel biomarkers for treatment of RC (21). Fortunately, emerging evidence has demonstrated that lncRNAs play a key role in the progression of RC, which may function as new targets for the development of therapeutic strategy for RC (22–24). In this paper, our aim is to investigate the functional role of lncRNA *IRAIN* in RC. After functional experiments and analysis were carried out, we found that overexpressed lncRNA *IRAIN* could inhibit cell proliferation and migration and induce cell apoptosis in RC by inhibiting VEGFA.

In this study, we show that lncRNA *IRAIN* was downregulated in RC cells and tissues, which was consistent with the results in RCC tissues and cells reported by Wang et al. (25). Similarly, a study also showed downregulation of lncRNA *IRAIN* in non-M3 acute myeloid leukemia clinical specimens (26). Our data also demonstrated that lncRNA *IRAIN* downregulated VEGFA via recruitment of DNA methylation to the promoter region of *VEGFA* as evidenced by increased recruitment of Dnmt1, Dnmt3a, and Dnmt3b at the *VEGFA* promoter after overexpression of lncRNA *IRAIN*. DNA methylation is capable of regulating gene expression, genomic stability, and cell fate commitment (27). This mechanism consists of two major processes: *de novo* methylation and maintenance of methylation, both of which are catalyzed by specific DNA methyltransferases including Dnmt1, Dnmt2, Dnmt3A, and Dnmt3b (28). Interestingly, DNA methylation of the *VEGFA* promoter by Dnmt3a-3L sc mutation results in inhibition of VEGFA (29). *VEGFA* is upregulated in a variety of cancers such as in osteosarcoma (30), breast cancer (31), and bladder cancer (32). A previous study had suggested that *VEGFA* is also upregulated in RC (33, 34). A similar result was reported in the current study that *VEGFA* was observed to have high expression in RC cells. Therefore, we concluded that lncRNA *IRAIN* decreased VEGFA expression by recruiting DNA methylases to the promoter region of *VEGFA*, thus playing a suppressive role in RC.

More importantly, lncRNA *IRAIN* overexpression repressed cell proliferation and migration but induced cell apoptosis in RC by downregulating VEGFA. A plethora of research studies identified that lncRNAs could target VEGFA in cancers such as lung adenocarcinoma (35) and endometrial carcinoma (36). LncRNA *IRAIN* stimulates anti-apoptosis and proliferation via methylation-dependent repression of Kruppel-like factor 2 and P15 in pancreatic cancer cells (11). Our findings were in support of the current literature, which clarified that upregulation of lncRNA *IRAIN* transcription triggered repression of cell proliferation, migration, and invasion in breast cancer cells (9). It is common knowledge that VEGFA has tumor-promoting properties by facilitating cell proliferation and migration (37–39). A corroborating study previously confirmed that suppression of VEGFA inhibits proliferation, migration, and promotes apoptosis of ccRCC cells (40). Another study also elucidated that inhibition of VEGFA as well as its receptor and the signal transduction pathway can inhibit tumor growth in cervical cancer (41). Consistent with our work, downregulation of VEGFA was shown to restrain cell proliferation and migration as well as facilitate cell apoptosis of endometrial cancer cells (36). More importantly, previous studies have pointed out the significance of methylation levels at the paraoxonase 1 (PON1) and forkhead box protein M1 (FOXM1) promoter regions to the progression and development of RC (42, 43), highly suggestive of the potential regulatory function of lncRNA *IRAIN* in the progression and development of RC involving other protein targets like PON1 and FOXM1. Also, *VEGFA* has been recognized to participate in tumorigenesis by mediating angiogenesis (44, 45), yet we focused on the regulatory role of *VEGFA* in RC cell survival, migration, and invasion, underscoring the various forms of regulation of *VEGFA* in RC, which requires further investigation in the future. However, given the mounting evidence regarding the aberrant expression of VEGFA in RC (40, 46), treatment modalities involving antibodies against VEGFA may hold therapeutic promise for RC management.

In summary, our study supports the anti-oncogenic potential of lncRNA *IRAIN* in RC cells and provides evidence that upregulation of lncRNA *IRAIN* or downregulation of *VEGFA* could suppress RC cell growth by inhibiting cell proliferation, migration, and stimulation of cell apoptosis. Therefore, investigating the lncRNA *IRAIN* and its functions could offer a better understanding of RC regulatory mechanisms, which may highlight a conserved therapeutic target against RC. Nevertheless, investigation of a specific mechanism of lncRNA *IRAIN* in other subtypes of RC remains to be done.

## Data Availability Statement

The datasets generated for this study are available on request to the corresponding author.

## Ethics Statement

Experiments involving animals were performed in accordance with the recommendations in the Guide for the Care and Use of Laboratory Animals of the National Institutes of Health and under the approval of the Ethics Committee of Huaihe Hospital, Henan University. The study was conducted under the approval of the Ethics Committee of Huaihe Hospital, Henan University.

## Author Contributions

YL, QL, and ZL designed the study. YW and CZ collated the data, carried out the data analyses, and produced the initial draft of the manuscript. TL and XL contributed to drafting the manuscript. All authors have read and approved the final submitted manuscript.

## Conflict of Interest

The authors declare that the research was conducted in the absence of any commercial or financial relationships that could be construed as a potential conflict of interest.

## Supplementary Material

Supplementary Figure 1 Upregulated lncRNA *IRAIN* curbs A498 cell proliferation and migration in contribution to A498 cell apoptosis through downregulation of VEGFA. The A498 cells were transfected with oe-NC, oe-*IRAIN* or oe-*IRAIN* + oe-*VEGFA*. **(A)** The expression of lncRNA *IRAIN* in A498 cells examined by RT-qPCR. **(B)** The expression of VEGFA in A498 cells determined by Western blot analysis normalized to GAPDH. **(C)** Proliferation of A498 cells measured by CCK-8 assay. **(D)** Migration of A498 cells examined by Transwell assay (×200). **(E)** Protein levels of EMT-related factors (E-cadherin and N-cadherin) in A498 cells normalized to GAPDH determined by Western blot analysis. **(F)** Apoptosis of A498 cells detected by flow cytometry. **(G)** The expression of apoptosis-related factors (bcl-2, Bax and cleaved-caspase3) measured by Western blot analysis normalized to GAPDH. ^*^*p* < 0.05 A498 cells transfected with oe-*IRAIN vs*. A498 cells transfected with oe-NC. ^#^*p* < 0.05 A498 cells transfected with oe-*IRAIN* + oe-*VEGFA vs*. A498 cells transfected with oe-*IRAIN*. Measurement data were expressed as mean ± standard deviation. Comparisons among multiple groups were conducted by one-way ANOVA with Tukey's *post-hoc* test. Data at different time points were compared by repeated measures ANOVA, followed by Bonferroni *post-hoc* test. The experiment was repeated independently 3 times.

## Abbreviations

lncRNA: long non-coding RNA
RC: renal carcinoma
VEGFA: vascular endothelial growth factor A
RCC: renal cell carcinoma
ccRCC: clear cell RCC
TNM: tumor-node-metastasis
RT-qPCR: reverse transcription quantitative polymerase chain reaction
NC: negative control
*GAPDH*: glyceraldehyde-3-phosphate dehydrogenase
IgG: immunoglobulin G
WT: wild type
MUT: mutant type.
