# Supplementary figures and images for: Long Non-coding RNA IRAIN Inhibits VEGFA Expression via Enhancing Its DNA Methylation Leading to Tumor Suppression in Renal Carcinoma
Source: Front Oncol. 2020 Sep 2;10:1082. doi: 10.3389/fonc.2020.01082 (PMC7492562; doi:10.3389/fonc.2020.01082)

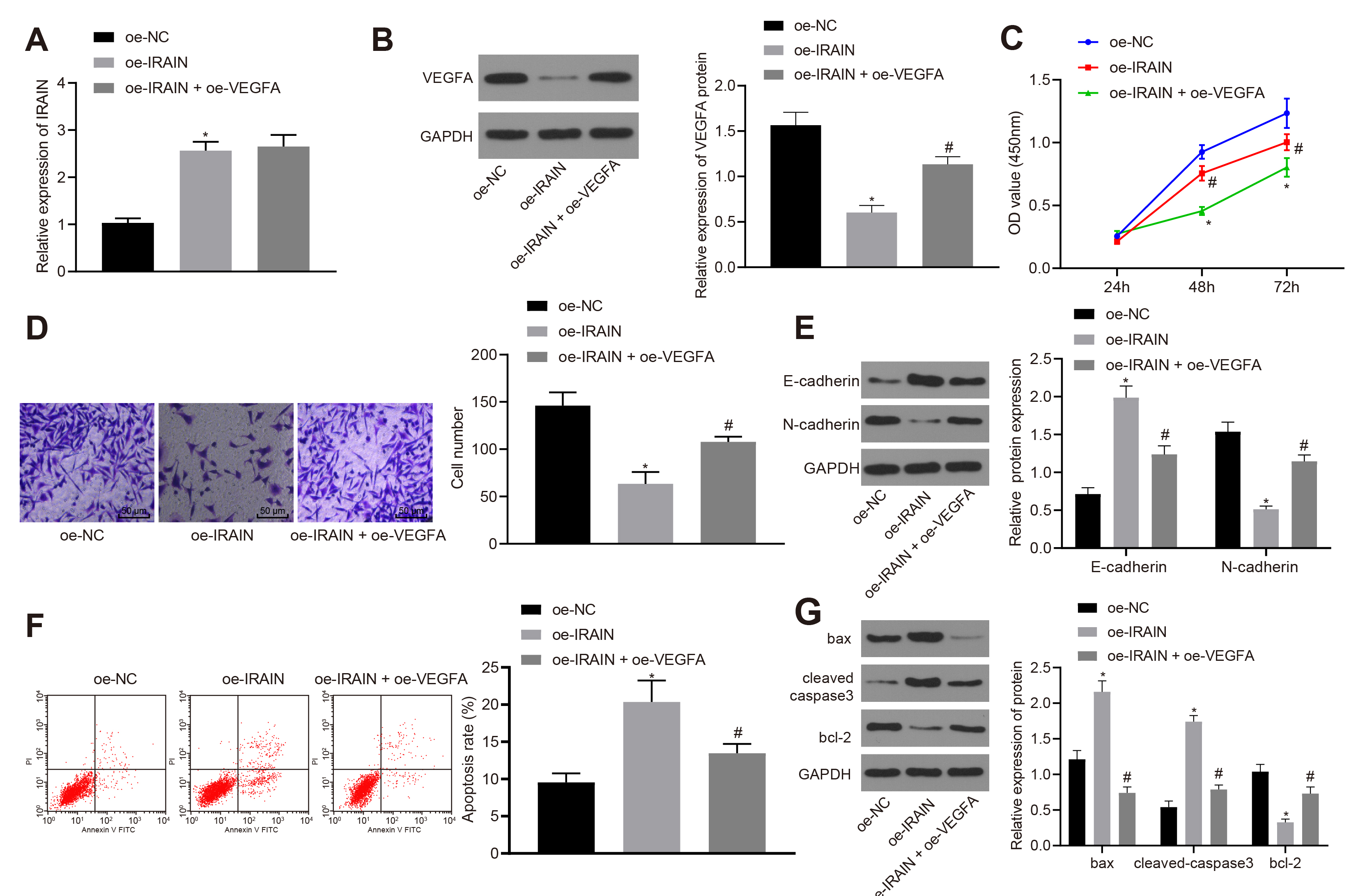

Supplement: Supplementary Figure 1 — Upregulated lncRNA IRAIN curbs A498 cell proliferation and migration in contribution to A498 cell apoptosis through downregulation of VEGFA. The A498 cells were transfected with oe-NC, oe-IRAIN or oe-IRAIN + oe-VEGFA. (A) The expression of lncRNA IRAIN in A498 cells examined by RT-qPCR. (B) The expression of VEGFA in A498 cells determined by Western blot analysis normalized to GAPDH. (C) Proliferation of A498 cells measured by CCK-8 assay. (D) Migration of A498 cells examined by Transwell assay (×200). (E) Protein levels of EMT-related factors (E-cadherin and N-cadherin) in A498 cells normalized to GAPDH determined by Western blot analysis. (F) Apoptosis of A498 cells detected by flow cytometry. (G) The expression of apoptosis-related factors (bcl-2, Bax and cleaved-caspase3) measured by Western blot analysis normalized to GAPDH. *p < 0.05 A498 cells transfected with oe-IRAIN vs. A498 cells transfected with oe-NC. #p < 0.05 A498 cells transfected with oe-IRAIN + oe-VEGFA vs. A498 cells transfected with oe-IRAIN. Measurement data were expressed as mean ± standard deviation. Comparisons among multiple groups were conducted by one-way ANOVA with Tukey's post-hoc test. Data at different time points were compared by repeated measures ANOVA, followed by Bonferroni post-hoc test. The experiment was repeated independently 3 times. [file Image_1.JPEG]
